# Degradable Starch Microspheres Transarterial Chemoembolization with or without Lipiodol for Liver Metastases from Pancreatic Cancer: A Prospective Randomized Trial

**DOI:** 10.3390/cancers15215239

**Published:** 2023-10-31

**Authors:** Thomas J. Vogl, Charlotte Lilienthal, Tatjana Gruber-Rouh, Zahra Afraz, Hamzah Adwan

**Affiliations:** Department of Diagnostic and Interventional Radiology, University Hospital, Goethe University Frankfurt, Theodor-Stern-Kai 7, 60590 Frankfurt, Germany; charlottefohr@web.de (C.L.);

**Keywords:** liver metastases, pancreatic cancer, transarterial chemoembolization, degradable starch microspheres, Lipiodol

## Abstract

**Simple Summary:**

The treatment of malignant liver tumors can be very challenging, especially in cases of metastatic disease. Systemic chemotherapy is most commonly used in such cases. However, liver metastases can also be treated by various interventional treatments including thermal ablation or TACE, depending on the hepatic tumor burden. This prospective, randomized study aims to compare DSM-TACE with Lipiodol + DSM TACE for patients with liver metastases from pancreatic cancer according to the volume and diameter of metastases, ADC values, and survival time of both groups after treatment of three TACE sessions. The treatment response of both cohorts will be assessed using RECIST 1.1.

**Abstract:**

To evaluate and compare the outcome of patients with liver metastases from pancreatic cancer treated by transarterial chemoembolization (TACE) using two different protocols. In this prospective, randomized, single-center trial, patients were randomly assigned to receive TACE therapy either with degradable starch microspheres (DSM) alone or a combination of Lipiodol and DSM. From the initial 58 patients, 26 patients (13 DSM-TACE, 13 Lipiodol + DSM-TACE) who completed 3 TACE treatments at an interval of four weeks were considered for evaluation of tumor responses. Initial and final MRIs were used to evaluate local therapy response by RECIST 1.1; changes in diameter, volume, ADC value, and survival rate were statistically evaluated. The differences between the DSM-TACE and Lipiodol + DSM-TACE were identified for partial response (PR) as 15.4% versus 53.8%, stable disease (SD) as 69.2% versus 46.2%, progressive disease (PD) as 15.4% versus 0%, respectively (*p* = 0.068). Median overall survival times for DSM-TACE and Lipiodol + DSM-TACE were 20 months (95% CI, 18.1–21.9) and 23 months (95% CI, 13.8–32.2), respectively (*p* = 0.565). The one-year survival rates for DSM-TACE and Lipiodol + DSM-TACE were 85.4% and 60.4%, the two-year survival rates were 35.9% and 47.7%, and the three-year survival rates were 12% and 30.9%, respectively. The evaluated local therapy response by RECIST 1. was not significantly different between the two studied groups. A longer overall survival time was observed after Lipiodol + DSM-TACE therapy; however, it was not significantly different.

## 1. Introduction

**Figure 1:**
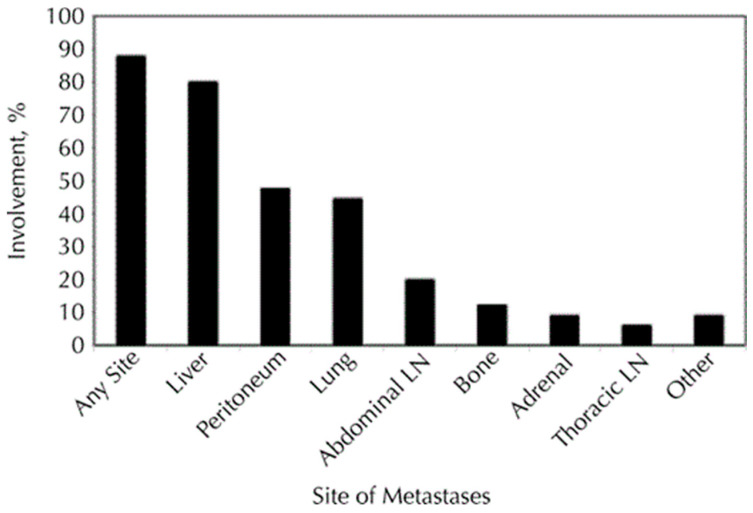
Involvement of metastatic infiltration by pancreatic cancer with different organ locales. The figure illustrates that the liver is the most prevalent body region of metastases for pancreatic cancer. These figures originate from the research conducted by Yachida et al. [7].

Pancreatic cancer is an aggressive solid malignancy with an increasing diagnosis rate over the previous years. Risk factors for pancreatic cancer are age, overweight, smoking, alcohol abuse, chronic pancreatitis, family history, and prior abdominal radiotherapy [1,2]. Different types of cancer affecting the pancreas are ductal adenocarcinoma, cystadenocarcinoma, and other cancers, including sarcomas and metastases. The most frequent type of pancreatic cancer is adenocarcinoma, with 90% of all diagnosed cases and the lowest 5-year survival rate [2,3,4]. Most patients with pancreatic cancer remain asymptomatic and therefore are diagnosed at an advanced stage later on [5]. Around 50% of patients with pancreatic cancer have distant metastases at the time of diagnosis, most of which appear in the liver [3,4]. At the time of diagnosis, about 35% of pancreatic cancers are identified with unresectable locally advanced pancreatic cancer (LAPC) [6]. Figure 1 shows the percentage distribution of metastatic locations in pancreatic cancer [7]. Less than 20% of patients could benefit from the curative resection surgery [8,9]. Patients in the unresectable stages of the disease, including the ones with distant metastases, could benefit from palliative and supportive therapy options [8].

Liver neoplasms are mainly supplied through an arterial flow and 95% by the hepatic artery [10,11]. The portal circulation of the liver usually supplies healthy hepatocytes [10,12]. Transarterial chemoembolization (TACE) is a catheter-based, minimally invasive method that locally targets the arteries supplying the liver lesion. TACE could be used for palliative or supportive therapy or as an adjuvant therapy option [13]. Direct administration of the chemotherapeutic agents through the catheter enables higher local doses compared to systematic chemotherapy, with far less complication compared to systemic therapy, including toxicity [11]. 

The subsequent embolization also extends the local presence of the chemotherapeutic agent within the tumor [13]. Hypoxia after arterial embolization increases the vascular permeability in the affected area, leading to higher cellular and tissue permeability to the cytostatic drug into and within the targeted liver metastases. Ultimately, the embolization induces ischemia and cellular death [14]. 

Degradable starch microspheres (DSM) could cause intra-arterial occlusion. Hence, coadministration of a cytostatic drug and DSM leads to selective enrichment in the small arteries leading to liver tumors [15]. Lipiodol is an oil-based contrast medium consisting of different fatty acids used as an embolization agent. Lipiodol diffuses through the liver sinusoids and can occlude arterial and portal venous circulation. Arterial blood is mainly exclusive for perfusing encapsulated liver tumors. However, infiltrative lesions and satellite nodules are supplied with portal blood via sinusoids. Therefore, targeting both blood pathways can be favorable. Most embolic agents predominantly obstruct the hepatic artery system and are ineffective in occluding the portal vein system [16].

Until now, very few studies have compared the effect of DSM as an embolization agent and the combination of Lipiodol and DSM in TACE therapy for pancreatic carcinoma with liver metastasis (PCLM). Therefore, this prospective, randomized comparative study aims to compare treatment response using Response Evaluation Criteria in Solid Tumors (RECIST 1.1) classification, tumor volume, tumor diameter, survival time, necrotic area and ADC value.

## 2. Materials and Methods

### 2.1. Study Design

This prospective, randomized comparative study was carried out at the radiological institute of the University Hospital Frankfurt. Institutional review board approval was obtained before the trial, and all patients offered their informed consent prior to participation in the study. 

Fifty-eight patients with pancreatic adenocarcinoma with liver metastases who were candidates for repeated TACE therapy were included in this study. A complete list of inclusion and exclusion criteria is provided in the next section. The clinical study center performed randomization and patient allocation.

Prior therapies consisted of systemic chemotherapy, radiation therapy and surgery. 

As a prerequisite for use in TACE treatment, cytostatics should exhibit an elevated hepatic extraction, short plasma half-life as well as effectiveness in hypoxic tissue. A combination chemotherapy regimen composed of Mitomycin C (Medac^®^, Hamburg, Germany), Gemcitabine (Gemzar^®^, Lilly Pharma, Giessen, Germany), and Cisplatin (Teva^®^, Gry Pharma GmbH, Germany) fulfilled these criteria and was used for all patients in this study. Study participants were allocated to two groups based on the embolization method used by TACE therapy. Group DSM-TACE received embolization agent DSM (EmboCept^®^S, PharmaCept, Berlin, Germany), while the group Lipiodol + DSM-TACE obtained a combination of Lipiodol (Lipiodol Ultra-Fluid^®^, Guerbet, Villepinte, France) plus DSM. 

Patients were planned to have three TACE therapy sessions. Therapy response was controlled between every TACE session by magnetic resonance imaging (MRI) with diffusion imaging (without contrast medium) at an interval of 4 weeks. Perfusion imaging (with contrast medium) was performed before the first therapy and after the final treatment. If patients presented with two metastases, these were considered in the statistical evaluation. In cases where patients had only one metastasis, solely this reference metastasis was assessed. 

### 2.2. Inclusion and Exclusion Criteria

Inclusion criteria were providing informed consent, age > 18, liver dominant metastasis, diagnosis of pancreatic cancer with liver metastasis confirmed by histological and/or radiological examination, no response to systemic therapy, ability to undergo MRI examination on 1.5 or 3 tesla scanners, initial tumor size of >1 cm and a clinically indicated treatment with TACE. 

Exclusion criteria were contraindications for an MRI examination such as an incompatible pacemaker or metal implants, pregnancy or breastfeeding women, renal insufficiency stage 4 and 5, second carcinoma, severe allergic reaction to contrast medium, contraindication for TACE including poor health condition, ascites, tumor involvement over 70% of liver volume, advanced liver dysfunction, complete thrombosis of the portal vein.

### 2.3. Treatment Protocol

A local inguinal anesthetic was applied after initial sterilization and draping of the inguinal area. The common femoral artery was punctured using various catheters (such as 5F pigtail catheter, 5F sidewinder, 2.8.F coaxial microcatheter). An exploratory view of the abdomen and the celiac trunk was captured using a contrast medium and arteriography, followed by catheterization of the hepatic artery and the tumor-supplying artery. Chemotherapeutic agents (Gemcitabine 1000 mg/m^2^ body surface area, Cisplatin 35 mg/m^2^ body surface area, and Mitomycin 8 mg/m^2^ body surface area) were then injected into the target artery. Afterward, embolization was achieved either using DSM (size of 200 µm, 200–400 mg) or by a combination of Lipiodol (maximum 10 mL/m^2^ per body surface) and DSM. 

### 2.4. Imaging 

Magnetic resonance imaging (MRI) was used to evaluate the characteristics of the metastatic lesions, like volume and diameter, presence of necrosis, and apparent diffusion coefficient (ADC). MRI scanners (1.5T or 3T) were used for the study. Patients were placed on the gantry in a supine position and were scanned in a head-first entry direction. When indicated, a contrast medium (gadobutrol, one mmol/mL Bayer Vital GmbH, Leverkusen, Germany) was administered intravenously (0.1 mL/kg) with an injection rate of 1–2 mL/s—the access line was then rinsed after that with an injection of 3 mL isotonic saline solution (0.9%, Fresenius Kabi, Bad Homburg, Germany).

The sequences used for initial and final MRI were (Table 1): localizer, T2-weighted coronal and axial with and without fat saturation, and T1-weighed FLASH 2D axial unenhanced and enhanced with contrast media, diffusion-weighted imaging (DWI) (b50, b400, b800) axial, perfusion in T1-weighted 3D sequence with fat saturation in axial unenhanced as well as enhanced in arterial phase, portal venous phase, and late venous phase.

Before each TACE therapy session, a control MRI was performed using the following sequences: localizer, T2-weighted coronal and axial with and without fat saturation, and T1-weighed FLASH 2D axial unenhanced and enhanced with contrast media, diffusion-weighted imaging (DWI) (b50, b400, b800) axial.

### 2.5. Statistics

For statistical analysis, IBM SPSS Statistics (Version 29.0) was used. Quantitative variables such as volume, necrotic area, diameter, and diffusion parameters were expressed as mean ± SD, range. Normally distributed parameters were tested with a two-tailed *t* test. Non-normally distributed parameters were analyzed with the Mann-Whitney U test. Categorical data were expressed as frequencies and percentages. Chi-square tests were used to test categorical variables for differences. Correlations between two parameters were analyzed using Spearman correlation. The Kaplan-Meier estimator was used for survival data. The logrank test was used to detect differences between survival data in the Lipiodol + DSM-TACE and DSM-TACE groups. For all analyses, a *p* ≤ 0.05 was assumed to be significant.

## 3. Results

### 3.1. Population

Initially, fifty-eight patients (26 females, 32 males) were included in the study. The clinical study center randomized the distribution into two groups. Patients were assigned to the DSM-TACE group or Lipiodol + DSM-TACE group based on the planned embolization method during TACE therapy. After fulfilling the inclusion and exclusion criteria and randomization, 30 participants were enrolled in the DSM-TACE group and 28 in the Lipiodol + DSM-TACE group.

On average, the patients were 61.3 years old (25–83 years). The average age for DSM-TACE and Lipiodol + DSM-TACE groups was 61 and 61.7 years, respectively.

The features of both study cohorts are presented below (Table 2).

**Figure 2:**
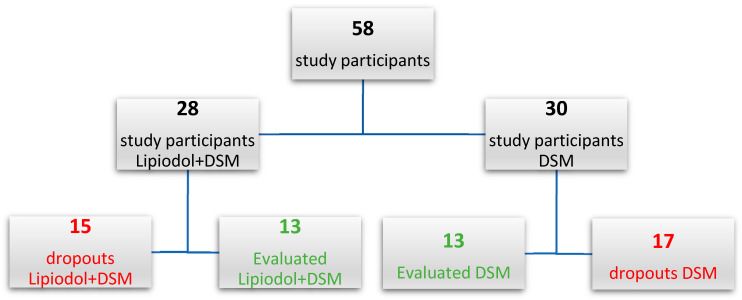
Randomization of study participants.

The number of participants who did not finish the treatment (dropouts) was 17 (56.7%) and 15 (53.6%) among DSM-TACE and Lipiodol + DSM-TACE groups, respectively. Figure 2 presents the randomization of both study groups.

The final results included participants who completed the study protocol (13 DSM-TACE, 13 Lipiodol + DSM-TACE). All fifty-eight participants were considered for the survival time analysis. Below, the study results are displayed in a tabular format (Table 3).

The total number of metastases among 26 final study participants was 47. The number of participants who had at least two metastases in DSM-TACE and Lipiodol + DSM-TACE was 12 and 9, respectively. All 47 metastases were included in statistical analysis.

### 3.2. Diameter

**Figure 3:**
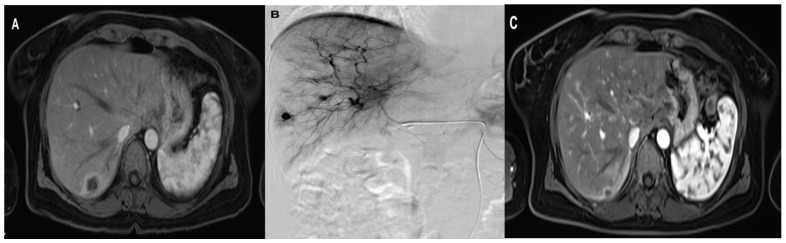
A 47-year-old woman with solitary liver metastasis in the right liver lobe treated with three sessions of Lipiodol + DSM TACE with Gemcitabine, Cisplatin, and Mitomycin. (**A**) pre-treatment axial MRI (**B**) During TACE (**C**) post-treatment axial contrast-enhanced MRI shows the significant downsizing of the metastasis.

The initial and final mean diameters of the metastatic lesions in Lipiodol + DSM-TACE were 4.5 cm (SD ± 2, range 1.4–7.8 cm) and 3.5 cm (SD ± 2, range 0.9–7.5 cm), respectively (Table 3). The Wilcoxon signed-rank test confirmed a significant metastases diameter difference in Lipiodol + DSM-TACE with an average diameter reduction of 25% (*p* = 0.002). Figure 3 demonstrates an exemplary treatment course of group Lipiodol + DSM-TACE.

The initial and final mean metastasis diameter for the DSM-TACE group was 4.0 cm (SD ± 1.5, range 1.2–7.1 cm) and 3.1 cm (SD ± 1.2, range 1.9–5.4 cm). The diameter reduction for metastatic lesions after the DSM-TACE therapy was significant, 16.4% on average (*p* = 0.033).

The initial metastasis diameter was not significantly different between the two groups (*p* = 0.650). Likewise, the two study groups showed no significant difference in final metastasis diameter (*p* = 0.614). The slightly higher diameter reduction in group Lipiodol + DSM-TACE compared to DSM-TACE was not statistically significant (*p* = 0.545).

### 3.3. Volume

The mean initial and final volume in the group Lipiodol + DSM-TACE was 24.1 cm^3^ (SD ± 27, range 2.3–84.2 cm^3^), and 12 cm^3^ (SD ± 17.8, range 1–66.8 cm^3^), respectively (Table 3). This group’s metastases volume was significantly reduced after the therapy (*p* = 0.005).

Similarly, the volume reduction after therapy with DSM-TACE was significant (*p* = 0.028). The initial and final mean volume in the DSM-TACE group was 10.7 cm^3^ (SD ± 10, range 0.9–33.8 cm^3^) and 6.3 cm^3^ (SD ± 6.3, range 1.3–20.9 cm^3^).

On average, the metastases volume reduction in the Lipiodol + DSM-TACE and DSM-TACE groups were 49.7% and 17.1%, respectively. The higher tumor volume reduction in group Lipiodol + DSM-TACE was not statistically significant (*p* = 0.390).

The initial metastasis volume was not significantly different between both groups (*p* = 0.139). Likewise, the final tumor volume measurement did not significantly differ between study groups (*p* = 0.479).

### 3.4. Necrotic Area

For evaluating the necrotic area, the longest diameter was measured for the study. The initial and final diameters of the necrotic area were analyzed. Three participants presented new necrotic lesions (two in group DSM-TACE and one in Lipiodol + DSM-TACE). These necrotic lesions presented after starting the trial were not included in the statistical analysis.

The initial MRI detected 13 necrotic lesions in 25 measured metastases in the DSM-TACE group. The mean diameter of the initial necrotic areas was 1.8 cm (SD ± 0.7 range 0.8–2.7 cm). The initial MRI in group Lipiodol + DSM-TACE located 11 necrotic areas in 22 measured metastases with a mean diameter of 1.8 cm (SD ± 1, range 0.8–3.6 cm).

Eight patients reached a complete regression of necrotic lesions during the therapy in both groups. The descriptive analysis of the final necrotic area also contains the complete remissions. The mean final diameter of the necrotic area in the Lipiodol + DSM-TACE and DSM-TACE groups was 0.5 cm (SD ± 0.8, range 0–1.7 cm) and 0.4 cm (SD ± 0.6, range 0–1.5 cm), respectively.

The diameter of the necrotic area was reduced by 76.2% and 68% in the Lipiodol + DSM-TACE and DSM-TACE groups, respectively. However, the greater size reduction in the necrotic area in group Lipiodol + DSM-TACE was insignificant (*p* = 0.721).

### 3.5. ADC

The mean value of the initial and final apparent diffusion coefficient (ADC) in the Lipiodol + DSM-TACE group was 2.1 × 10^−3^ mm^2^/s and 2.5 × 10^−3^ mm^2^/s (Table 3). On average, the ADC value increased significantly by 20% (*p* = 0.011).

**Figure 4:**
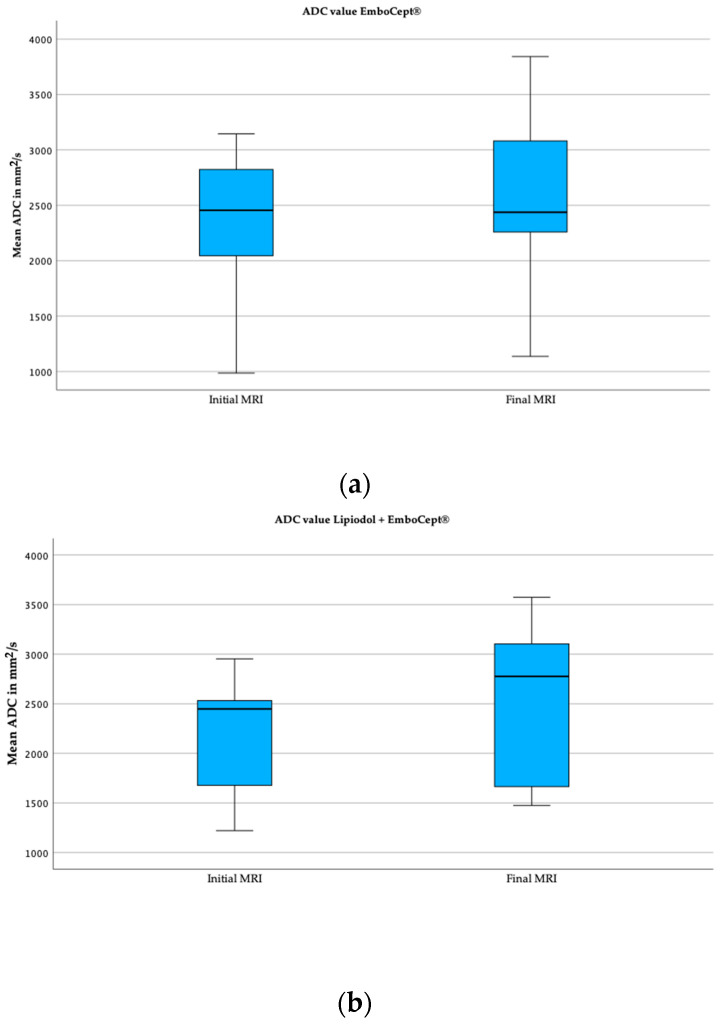
Boxplots of mean ADC values of both groups. (**a**) These boxplots capture the initial and final mean ADC value in group DSM. (**b**) These boxplots demonstrate the ADC value in group Lipiodol + DSM-TACE.

The initial and final mean values measured for ADC in the DSM-TACE group were 2.4 × 10^−3^ mm^2^/s and 2.6 × 10^−3^ mm^2^/s, respectively. However, the 10.2% increase between the initial and final ADC values was statistically insignificant (*p* = 0.064). Exemplary boxplots illustrating the initial and final ADC values for both cohorts are displayed below in Figure 4.

The mean values of the initial ADC (*p* = 0.336) and final ADC (*p* = 0.880) did not significantly differ between study groups. Even though the increased rate of the ADC value was higher in the Lipiodol + DSM-TACE group compared to DSM-TACE, it was not significantly different based on the Mann–Whitney U Test (*p* = 0.880).

There was no significant correlation between the increase in ADC values and tumor response based on metastases volume reduction (rho = −0.286, *p* = 0.156), diameter reduction (rho = −0.313, *p* = 0.120), or RECIST 1.1 (rho = 0.270, *p* = 0.182).

### 3.6. RECIST 1.1

The tumor response was evaluated by Response Evaluation Criteria in Solid Tumors 1.1 (RECIST 1.1). The RECIST 1.1 classifications for DSM-TACE versus Lipiodol + DSM-TACE showed no complete response, partial response in two (15.4%) versus seven (53.8%) cases, stable disease in nine (69.2%) versus six (46.2%) cases and progressive disease in two (15.4%) versus zero for Lipiodol + DSM-TACE group. There was no difference between the two groups based on the chi-square test (*p* = 0.068). The tumor response is presented in a Table 4 below.

There was a negative correlation between RECIST 1.1 and diameter reduction (rho= −0.801, *p* < 0.001) as well as volume reduction (rho = −0.604, *p* < 0.001). Hence, a better therapy response estimated by RECIST 1.1 is correlated with a higher reduction in the volume and diameter of the metastatic lesions.

### 3.7. Survival Analysis

All 58 participants were included in the survival analysis by Kaplan–Meier estimator. In Lipiodol + DSM-TACE and DSM-TACE groups, 16 and 19 participants died during or after the study period, respectively. The last follow-up date was considered for the survival analysis of the remaining 12 participants in the Lipiodol + DSM-TACE group and 11 patients in the DSM-TACE group. The median overall survival among all participants was 21 months (95% confidence interval (CI), 17–24).

**Figure 5:**
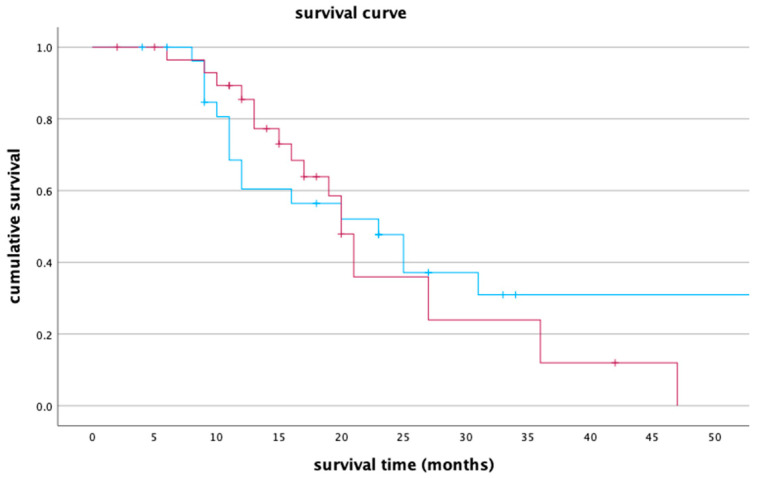
A survival curve comparing group Lipiodol + DSM-TACE (blue line) and group DSM-TACE (red line). The *x*-axis represents survival time in months, while the *y*-axis denotes cumulative survival.

The median survival time for Lipiodol + DSM-TACE and DSM-TACE groups was 23 months (95% CI, 13.8–32.2) and 20 months (95% CI, 18.1–21.9), respectively. Based on the log rank, both groups had no significant differences (*p* = 0.565). The survival curve for both treatment groups is presented below in Figure 5.

The one-year survival rate for Lipiodol + DSM-TACE and DSM-TACE groups was 60.4% (95% CI, 41.2–79.6) and 85.4% (95% CI, 72.1–98.7), respectively. The two-year survival rate for Lipiodol + DSM-TACE was 47.7% (95% CI, 27.9–67.5) versus 35.9% (95% CI, 14.5–57.3) in the DSM-TACE group. After three years, 30.9% (95% CI, 10.7–51.1) of the patients in the Lipiodol + DSM-TACE group were alive compared to only 12% (95% CI, 0.0–27.3) in DSM-TACE group.

## 4. Discussion

The results of this study show that using a combination of Lipiodol and DSM as the embolizing material for TACE therapy offers therapeutic advantages compared to using DSM alone. However, no significance was established between both groups.

A significant reduction in both the volume and diameter of the metastatic lesions was observed in both groups. Patients with better therapy responses based on their RECIST 1.1 score were shown to have higher volume and diameter size reduction. However, no significant difference was shown between DSM-TACE and Lipiodol + DSM-TACE regarding the metastatic lesions’ initial (4 cm vs. 4.5 cm) and final (3.1 cm vs. 3.5 cm) size. One possible explanation might be that the patients in both groups had similar and comparable characteristics.

In our study, no significant difference in survival time was found between the two study groups (*p* = 0.565). However, TACE with a combination of Lipiodol and DSM offered a longer survival time compared to DSM-TACE alone (23 months vs. 20 months).

A retrospective study of Vogl et al. examined tumor response and ADC patterns such as survival rates in cases of liver metastases from pancreatic adenocarcinoma. The study protocol closely resembled our study. All patients (*n* = 112) received the same chemotherapeutic agents and Lipiodol + DSM. The median survival for all patients was 19 months [17]. Our study demonstrates a median survival among all patients of 21 months. Similar to the study by Vogl et al., our study shows a significantly higher median survival using a triple-drug combination and Lipiodol + DSM compared to previous studies that used Cisplatin, Lipiodol and Gelfoam. With the same underlying condition and metastases, only a mean survival time of 9.6 months was achieved. This finding confirms the high effectiveness of our TACE therapy [17,18].

The study of Gruber-Rouh et al. on ninety-nine patients with hepatocellular carcinoma (HCC) treated with either a combination of Lipiodol + DSM-TACE or Lipiodol-TACE demonstrated a similar trend. In their study, there was a higher median survival time of 28 months in the Lipiodol + DSM-TACE group compared to 25 months without combined embolization in the Lipiodol-TACE group. Similar to our study, a slight advantage in tumor response and survival time for the combination therapy was also identified. However, it did not reach statistical significance [19].

A similar result to our study was observed in the study conducted by Azizi et al. Here, the same combination of cytostatics and the embolization agents Lipiodol + DSM were used for patients with the same underlying tumor condition. According to RECIST criteria, 71.87% showed SD, 9.37% had a PR and 18.75% had PD. A median survival of 16 months was observed. Among the patients with SD, the mean survival time extended to 20 months, compared to 5 months in those with PD [20]. In our Lipiodol + DSM-TACE group, no progression was observed, which likely contributed to a longer median survival time. In this cohort, we only observed stable disease and partial response. The analyzed median survival time of 23 months in this group seems plausible in comparison to the study mentioned earlier.

Further studies also found no significant difference in survival time when investigating two different embolization protocols. A study carried out by Niessen et al. examined local tumor response and overall survival between conventional TACE and TACE with degradable starch microspheres in 69 patients with unresectable intermediate-stage hepatocellular carcinoma (HCC). The mean survival did not significantly differ between both groups (*p* = 0.337) [21]. A similar outcome was also shown in a study by Vogl et al. This study compared the therapeutic response of TACE using Lipiodol or DSM in patients with colorectal liver metastases. The survival time after TACE using Lipiodol compared to DSM-TACE with 13 and 16 months was not significantly different between both groups (*p* = 0.75) [22].

Over recent years, the treatment for metastatic pancreatic carcinoma has been based on two chemotherapy combinations (FOLFIRINOX and Gemcitabin + nab-Paclitaxel) [23]. In the study by von Hoff et al., a median survival of 8.5 months was observed in the treatment group receiving nab-Pacliteaxel + Gemcitabine [24]. The 2011 study published by Conroy et al. reported a median overall survival of 11.1 months in the FOLFIRINOX treatment group [25].

A recently published meta-analysis in Japan conducted by Takumoto et al. examined the outcome of various first-line chemotherapies for metastatic pancreatic cancer. In the FOLFIRINOX group, the highest observed overall survival was 15.49 person-months. In the Gemcitabine + albumin-bound Paclitaxel group, an overall survival of 12.36 person-months was analyzed [26]. The considerably more precise administration of chemotherapeutic agents and embolic materials into the tumor-supplying artery through TACE favors an alternative to the previously mentioned first-line treatments. Through our treatment, we were able to achieve median survival times of 23 months in group Lipiodol + DSM-TACE and 20 months in DSM-TACE.

The initially elevated one-year survival rate in the DSM-TACE group, followed by the shifting trend towards a higher two- and three-year survival rate in the Lipiodol + DSM-TACE cohort may be attributed to various factors. It is plausible that the effectiveness of Lipiodol + DSM-TACE might have been less pronounced initially, but over time, it demonstrates a stronger impact. The response to therapy may have had a delayed effect in the Lipiodol + DSM-TACE group, which is why the two- and three-year survival rates are superior to those in the DSM-TACE group. Additionally, it can be considered that the course of cancer is often very complex and individual, and accordingly, each patient may respond differently to the TACE therapy.

In our study, local tumor response did not significantly differ between the two groups. Similarly, no significant difference between the tumor stages classified by RESIST 1.1 was shown between the two groups. However, the significance level was narrowly missed, with a *p*-value of 0.068. The combined therapy of both embolic agents exhibited an improved therapy outcome. Although nonsignificant, the improvement was more prominent in the group receiving a combination of Lipiodol and DSM for embolization.

A study by Vogl et al. investigated the difference between conventional TACE using Lipiodol and a combination of Lipiodol + DSM-TACE in patients with hepatocellular carcinoma (HCC). This study reported a more favorable outcome for the combination of Lipiodol and DSM. The tumor response evaluated by mRECIST was significantly improved in the combination therapy (*p* = 0.010). Likewise, the overall survival time observed in the Lipiodol-DSM group compared to the Lipiodol-TACE group was higher, although with no statistical significance (22.77 months versus 26.32 months, *p* = 0.844) [27]. This study was only partially comparable to our current study as different embolization protocols were used for each research project. While assessing various tumor responses between both groups, our present study merely narrowly missed the significance level. The tumor type studied by Vogl et al. was hepatocellular carcinoma. However, our current study evaluated aggressive pancreatic adenocarcinoma. The variation in the tumor type could justify a slight deviation in *p*-values between the two studies. However, the outcome was comparable to our study. The combined therapy using both embolic agents showed a promising tumor response and survival time outcome.

Research studies have shown that elevated ADC values were associated with a better response to chemotherapy [28]. Increased ADC values before chemotherapy were linked with a poor response to treatment [28,29]. Although an increase in ADC value was detected in group DSM-TACE and Lipiodol-DSM-TACE (10.2% vs. 20%), only group Lipiodol + DSM-TACE showed a significant difference between initial and final ADC value (*p* = 0.064 vs. *p* = 0.011). We, however, did not find any significant differences in correlation between higher ADC volume and diameter reduction or better local tumor response.

TACE is an established procedure that enables selective treatment by directly delivering chemotherapeutics agents into the tumor. Systemic side effects are reduced through direct administration in the tumor vascular network [27].

A limiting aspect of this study encompasses the restricted study population in both treatment groups. Owing to the severity of the disease, a few patients dropped out before the study was completed. As a result, the initially planned study population could not be achieved; consequently, this affects the integrity of our statistical analysis. Further studies should be designed with a larger sample size. For future studies, it might be worthwhile to incorporate a control arm that did not receive TACE therapy in order to assess the effectiveness of the treatment even more objectively. 

A study released by Das et al. analyzed diverse treatment modalities for advanced pancreatic cancer. It investigated whether the combination of different treatment methods has collaborative impact. It assessed if the combined interventional therapy (TACE combined with iodone-125 seed implantation and/or radiofrequency ablation), in comparison to TACE or chemotherapy alone, demonstrated greater efficacy. The combined interventional therapy showed strong effectiveness and an increased survival rate for non-operable pancreatic cancer in comparison to TACE alone [30]. A comparison of different TACE protocols in combination with ablation would be another interesting study for the future.

It would be also important to consider previous treatments and evaluate side effects as well as complications. That would lead to better comparability between both cohorts. Another constraining factor of this study was the exclusive implementation at a single study location. Future studies should be conducted at multiple study locations to establish better comparability and broaden the generalizability of the results.

## 5. Conclusions

This study demonstrated that TACE is a successful, selective, and palliative treatment of liver metastases in pancreatic cancer. While not statistically significant, the tumor response was slightly improved, and survival time was marginally prolonged in the group receiving Lipiodol-DSM. The combined usage of both embolic agents exhibited a beneficial treatment outcome.

## Figures and Tables

**Table 1:** Examination MRI protocol.

Examination Protocol (Initial and Final MRI)	Examination Protocol (Before Each TACE Therapy)
- Localizer in three planes - T2w-coronary and transverse with and without fat saturation - T1w-FLASH-2D transverse - EP-2D-Diff (b50, b400, b800) - T1w-3D native, transverse, with fat saturation - Contrast medium - Monitoring - Three dynamic T1w-3D transverse - T1w-FLASH-2D transverse	- Localizer in three planes - T2w-coronary and transverse with and without fat saturation - T1w-FLASH-2D transverse - EP-2D-Diff (b50, b400, b800)

**Table 2:** Characteristics of all study participants.

Characteristics	DSM-TACE Group	Lipiodol + DSM-TACE Group	Total	*p* Value
Number of patients	30	28	58	
Sex				0.771
Male	16 (53.3%)	16 (57.1%)	32 (55.2%)	
Female	14 (46.7%)	12 (42.9%)	26 (44.8%)	
Mean age (years)	61.0	61.7	61.3	0.913
Localization				0.793
Right liver lobe	4 (13.3%)	5 (17.9%)	9 (15.5%)	
Left liver lobe	2 (6.7%)	1 (3.6%)	3 (5.2%)	
Both lobes	24 (80%)	22 (78.6%)	46 (79.3%)	
Number of liver lesions				0.677
1 lesion	2 (6.7%)	2 (7.1%)	4 (6.9%)	
2 lesions	3 (10%)	5 (17.9%)	8 (13.8%)	
≥3 lesions	25 (83.3%)	21 (75%)	46 (79.3%)	

**Table 3:** Results.

Parameter	DSM-TACE Group (±SD) (*n* = 13)	Lipiodol + DSM-TACE Group (±SD) (*n* = 13)	*p* Value
Mean tumor volume			
Baseline	10.7 cm^3^ (±10)	24.1 cm^3^ (±27)	0.139
Endpoint	6.3 cm^3^ (±6.3)	12 cm^3^ (±17.8)	0.479
Mean tumor diameter			
Baseline	4 cm (±1.5)	4.5 cm (±2)	0.650
Endpoint	3.1 cm (±1.2)	3.5 cm (±2)	0.614
Tumor response (RECIST 1.1)			
Partial response (PR)	15.4% (*n* = 2)	53.8% (*n* = 7)	
Stable disease (SD)	69.2% (*n* = 9)	46.2% (*n* = 6)	0.068
Progressive disease (PD)	15.4% (*n* = 2)	-	
Mean ADC value			
Baseline	2.4 × 10^−3^ mm^2^/s	2.1 × 10^−3^ mm^2^/s	0.336
Endpoint	2.6 × 10^−3^ mm^2^/s	2.5 × 10^−3^ mm^2^/s	0.880

**Table 4:** Tumor response (RECIST 1.1).

Tumor Response (RECIST 1.1)	DSM-TACE Group (*n* = 13)	Lipiodol + DSM-TACE Group (*n* = 13)
Partial response (PR)	15.4% (*n* = 2)	53.8% (*n* = 7)
Stable disease (SD)	69.2% (*n* = 9)	46.2% (*n* = 6)
Progressive disease (PD)	15.4% (*n* = 2)	-
Complete response (CR)	-	-

## Data Availability

Data are available upon request from the corresponding author.
